# Quercetin Application for Common Carp (*Cyprinus carpio*): I. Effects on Growth Performance, Humoral Immunity, Antioxidant Status, Immune-Related Genes, and Resistance against Heat Stress

**DOI:** 10.1155/2023/1168262

**Published:** 2023-02-17

**Authors:** Kobra Armobin, Ehsan Ahmadifar, Hossein Adineh, Mahsa Naderi Samani, Naser Kalhor, Sevdan Yilmaz, Seyed Hossein Hoseinifar, Hien Van Doan

**Affiliations:** ^1^Department of Fisheries, Faculty of Natural Resources, University of Zabol, Zabol, Iran; ^2^Department of Fisheries, Faculty of Agriculture and Natural Resources, Gonbad Kavous University, Gonbad Kavous, Golestan, Iran; ^3^Iran Fisheries Organization, Tehran, Iran; ^4^Department of Mesenchymal Stem Cell, Academic Center for Education, Culture and Research, Qom Branch, Qom, Iran; ^5^Çanakkale Onsekiz Mart University, Department of Aquaculture, Faculty of Marine Science and Technology, 17100 Çanakkale, Turkey; ^6^Department of Fisheries, Faculty of Fisheries and Environmental Sciences, Gorgan University of Agricultural Sciences and Natural Resources, Gorgan, Iran; ^7^Department of Animal and Aquatic Sciences, Faculty of Agriculture, Chiang Mai University, Chiang Mai 50200, Thailand

## Abstract

This study was done to evaluate the effect of different quercetin levels on growth performance, immune responses, antioxidant status, serum biochemical factors, and high-temperature stress responses in common carp (*Cyprinus carpio*). A total number of 216 common carp with an average weight of 27.21 ± 53 g were divided into 12 tanks (four treatments × three replications) and fed 0 mg/kg quercetin (T0), 200 mg/kg quercetin (T1), 400 mg/kg quercetin (T2), and 600 mg/kg quercetin (T3) for 60 days. There were significant differences in growth performance, and the highest final body weight (FBW), weight gain (WG), specific growth rate (SGR), and feed intake (FI) were observed in T2 and T3 (*P* < 0.05). Different quercetin levels significantly increased complement pathway activity (ACH50) and lysozyme activity both before and after heat stress (*P* < 0.05). Catalase (CAT), glutathione peroxidase (GPx), and malondialdehyde (MDA) were significantly increased in fish exposed to heat stress, but fish fed with a supplemented diet with quercetin showed the lowest levels both before and after heat stress (*P* < 0.05). Superoxide dismutase (SOD) levels were significantly enhanced in fish fed diets supplemented with quercetin in both phases (*P* < 0.05). Different quercetin levels led to a significant decrease in alanine aminotransferase (ALT) and aspartate aminotransferase (AST) before and after the challenging test (*P* < 0.05). Glucose and cortisol levels were significantly higher in the control group compared to the other treatments in both phases (*P* < 0.05). The expression of glutathione peroxidase (GPx) and lysozyme was markedly upregulated in fish fed with quercetin-supplemented diets (*P* < 0.05). No marked effects were observed for growth hormone (GR) and interleukin-8 (IL8) (*P* > 0.05). In conclusion, dietary quercetin supplementations (400-600 mg/kg quercetin) improved growth performance, immunity, and antioxidant status and increased tolerance to heat stress.

## 1. Introduction

Aquaculture is developing incrementally in parallel with global population growth. Nevertheless, intensive production activities, sudden temperature changes, abnormal climate, and other environmental conditions as such cause stress to fish and hamper production quality and yield by triggering immune depression and increased infectious epidemics. One of the most important environmental items in the aquaculture industry is temperature because it can affect the metabolism, feeding behavior, growth performance, survival, and disease resistance of aquatic animals [1–3]. Temperature changes decreased the growth, feed efficiency, physiological function, immune response, and oxidative status in the organs of aquatic animals ([3–5].

To combat such challenges, the involvement of functional feed additives in the fish diet is environment-friendly and efficient. These additives can potentially increase the growth and reproductive performance of fish, fillet quality, and resistance to diseases and environmental conditions.

Polyphenols and polyphenol-rich additives are added to aquatic animal feed to increase growth performance, immune response, antioxidant status, and disease resistance [6, 7]. Being considered the main class of flavonoids (flavonols), a family of polyphenolic compounds, quercetin is abundant in many fruits and vegetables, including tea, onions, apples, strawberries, cabbage, nuts, and cauliflower.

It is widely acknowledged that quercetin inhibits biofilm production of many pathogenic bacteria such as *Enterococcus faecalis*, *Staphylococcus aureus*, *Streptococcus mutans*, *Escherichia coli*, and *Pseudomonas aeruginosa* with its features such as suppression of quorum-sensing pathways, inhibition of efflux pumps, disruption or alteration of the plasma membrane, and blocking nucleic acid synthesis [8]. Moreover, quercetin has many beneficial properties such as being an anticancer [9], antioxidant, and anti-inflammatory [10].

Previous findings indicated that dietary quercetin had a positive effect on the antioxidant status [11, 12], growth performance [13, 14], flesh quality [15], hematological and immune parameters [16], disease resistance [17, 18], and grouper iridovirus defense properties [19–21]. Some researches showed that dietary quercetin enhanced the growth performance of chicken [22] and boosted sensory scores such as color, texture, and overall acceptability of goat loin [23]. In aquatic animals, quercetin above 400 mg/kg improved the growth performance of tilapia (*Oreochromis* spp.) [24].

Previous studies have reported that dietary herbal supplementations reduce stress markers in some fish and/or shellfish species like *Macrobrachium rosenbergii* [25], *Megalobrama amblycephala* [26], and *Oncorhynchus mykiss* [27] subjected to high temperature. However, no information is available as to the effect of dietary quercetin on aquatic animals exposed to high-temperature stress. Therefore, the present study was carried out to compare the effect of dietary quercetin supplementations on growth performance, some immune parameters, antioxidant status, some serum biochemical variables, and high-temperature stress responses in fish.

## 2. Material and Methods

### 2.1. Experimental Diets

Four experimental diets with different quercetin (>95% purity, Sigma Chemical Co., USA) levels were formulated (Table 1). Briefly, the dry ingredients were mixed thoroughly. After the liquid ingredients were diffused into the mixture, deionized water was added (250 ml per kg of diet). The prepared mixture was extruded in an electric meat grinder (MG1400R, Pars Khazar, Tehran, Iran), and feeds were passed off based on the fish's mouth size (1 mm in diameter). Finally, four experimental diets were prepared including 0 mg/kg quercetin (T0), 200 mg/kg quercetin (T1), 400 mg/kg quercetin (T2), and 600 mg/kg quercetin (T3). The pellets were dried in a drying cabinet and then stored at 4°C until use. The chemical composition, moisture, crude protein, lipids, and ash contents were confirmed by following the standard methods [28].

### 2.2. Fish and Experimental Condition

A total number of 216 common carp with an average weight of 27.21 ± 53 g were obtained from a private fish farm and moved to the research hall of the University of Gonbad (Gonbad, Iran). They were stored in 12 tanks with a density of 18 fish per tank and acclimated to experimental conditions for 10 days. During the acclimation period, common carp were fed a basal diet twice a day. At the end of adaptation time, these tanks were randomly nominated to 4 treatments with 3 replicates, and fish were fed at a rate of 2.5% of body weight twice a day for 60 days. Fish waste and half of the aquarium water were siphoned daily and replaced with well-aerated water. Water quality parameters such as temperature, dissolved oxygen, and pH were regularly measured and kept at standard levels for common carp (temperature 24.1 ± 0.5C, dissolved oxygen 6.8 ± 0.2mg/l, pH7.36 ± 0.1, and photoperiod 12D : 12L) [29].

### 2.3. Biometry and Blood Sample Collection

After a 24 h fasting period, all fish in each tank were anesthetized using 200 mg l^−1^ clove powder, and the weight of each fish was recorded [30]. The growth performance and survival rate were calculated using a common formula [7]. Three fish per tank (9 fish per treatment) were randomly selected to collect the blood sample. Blood samples were collected via the caudal vein of each fish using a sterile syringe and introduced into tubes. The tubes were centrifuged (5000 rpm for 10 min at 4°C) [29], and then, the supernatant was separated and stored at -20°C.

### 2.4. Heat Challenge

After 60 days of feeding, a heat stress challenge was administered according to the Dawood et al. [30] study in triplicate. First, feeding was stopped during the test, and seven starved fish per tank (21 fish per treatment) were held at a similar condition with a high temperature (32°C) for 48 hours for heat stress. Aquarium heaters were used to increase the temperature up to 32°C, and the water temperature was checked frequently by portable multiparameter meters (YSI, China).

### 2.5. Blood and Tissue Sampling

After the challenging test, all fish in each tank were anesthetized using 200 mg l^−1^ clove powder, and three fish per tank (9 fish per treatment) were selected to collect blood samples. Blood samples were collected via the caudal vein of each fish using a sterile syringe and introduced into tubes. The tubes were centrifuged (5000 rpm for 10 min at 4°C) [29], and then, the supernatant was separated and stored at -20°C.

### 2.6. Biochemistry Assay

Before and after challenging tests, glucose, alanine aminotransferase (ALT), aspartate aminotransferase (AST), catalase (CAT), glutathione peroxidase (GPx), malondialdehyde (MDA), and superoxide dismutase (SOD) were assayed using commercial kits (Pars Azmoon, Iran). Serum lysozyme activity was assayed using the method of Saurabh and Sahoo [31] with turbidimetry. First, a suspension of *Micrococcus lysodeikticus* was prepared by dissolving 0.2 mg ml^−1^ in a 0.05 M sodium phosphate buffer (pH 6.2). Then, 50 *μ*l serum was added to a 2 ml suspension of *Micrococcus lysodeikticus*, and the reaction mixture was divided into a 96-well microtitre plate (Hiperion, Germany), and initial OD was measured spectrophotometrically at 450 nm immediately. The final OD was measured after incubation of the reaction mixture at 24°C for 1 h. A unit of lysozyme activity was defined as the sample amount causing a decrease in absorbance of 0.001/min lysozyme of the sample calibrated using a standard curve determined with hen's egg white lysozyme (Sigma) in PBS. Serum alternative complement (ACH50) activity was measured based on Yano et al. [32], in which rabbit red blood cells (RBCs) were used as a target. Serum cortisol levels were measured by the competitive ELISA method using a commercial kit (IBL Co., Gesellschaft für Immunchemie und Immunbiologie).

### 2.7. Gene Expression

Total RNA of fish livers collected after a challenging test was isolated using the GeneAll Kit (GeneAll Biotechnology, Seoul, Korea). After evaluating RNA quality by electrophoresis agarose gels, 2 *μ*g of total RNA was treated with DNase I and used for first-strand cDNA synthesis using oligo (dT) primers, 10 mM dNTPs, and reverse transcriptase according to the manufacturer's instructions (Thermo Scientific, Germany). Gene-specific primers were mentioned for RT-PCR analysis in Table 2. The beta-actin gene was used as an internal control. qPCR was carried out using RealQ Plus Master Mix Green (AMPLIQONIII) following the manufacturer's instructions. The reaction consisting of 10 *μ*l SYBR green mix, 1 *μ*l of diluted cDNA, 0.5 *μ*l of each primer, and Millipore water was added to a final volume of 20 *μ*l. The following program was used for the reaction: 10 min at 95°C, denaturation at 95°C for 10 s, annealing at 60°C for 40 s, and extension at 72°C for 35 s for 40 cycles. The RNA level was calculated based on the 2^-*ΔΔ*CT^ method [33]. Three biological repeats were done for each experiment.

### 2.8. Statistical Analysis

Data (mean ± SD) were analyzed by one-way analysis of variance (ANOVA) followed by Duncan's multiple range test to compare the means between groups. Before analyses, data were assessed for normality and homogeneity of variance with the Shapiro-Wilk test and Levene's test, respectively. Two-way ANOVA was also used to test the effects of the diet, heat stress, and their interactions. Statistical analyses were conducted using SPSS software version 23, and *P* < 0.05 was the accepted significance level.

## 3. Results

### 3.1. Growth Performance

There were significant differences in growth performance, and the highest FBW, WG, SGR, and FI were seen in T2 and T3 (Table 3, *P* < 0.05). FCR was significantly decreased in fish fed quercetin (Table 3, *P* < 0.05).

### 3.2. Serum Immune Response

Lysozyme and ACH50 activity were remarkably decreased when common carps were exposed to heat stress, and the lowest activity was observed in fish fed a control diet and fish fed with supplemented diets, representing the highest levels (Table 4, *P* < 0.05). Before and after heat stress, different quercetin levels significantly increased ACH50 and lysozyme activity, and the highest activities were seen in fish fed 400 (T2) and 600 (T3) mg/kg quercetin (Table 4, *P* < 0.05).

### 3.3. Antioxidant Enzymes

Catalase, MDA, and GPx were significantly raised in fish exposed to heat stress, with the highest level in fish fed with a control diet and fish fed with a supplemented diet with quercetin showing the lowest levels (Table 4, *P* < 0.05). SOD levels decreased significantly in fish exposed to heat stress (when all treatments were combined); also, there was a significant difference in the levels of SOD amongst the treatments before and after heat challenge, and the highest level was recorded in T2 in both phases (Table 5, *P* < 0.05).

### 3.4. Stress Parameters

Before and after heat stress, glucose and cortisol levels were significantly higher in the control group compared to quercetin supplementation treatments (Table 6, *P* < 0.05). Exposure of common carp to high temperatures led to a significant increase in AST and ALT in all treatments, but fish fed with 600 mg/kg quercetin had the lowest levels (Table 6, *P* < 0.05). Moreover, before and after the challenge, the highest and lowest activities of ALT and AST were seen in the control group and T3, respectively (Table 6, *P* < 0.05).

### 3.5. Gene Expression

**Figure 1:**
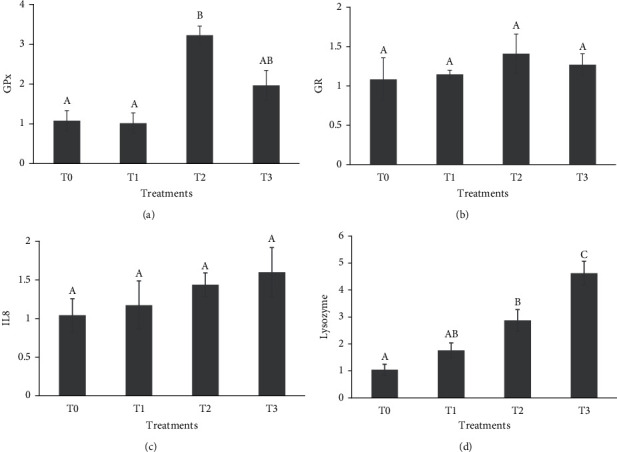
Relative expression of glutathione peroxidase (a, GPx), glutathione reductase (b, GR), interleukin-8 (c, IL8), and lysozyme (d) of common carp (*Cyprinus carpio*) fed with 0 (T0), 200 (T1), 400 (T2), and 600 (T3) mg/kg quercetin for 8 weeks and challenged with heat stress. Different letters designate significant differences as determined by Duncan's multiple range test (mean ± SD).

After heat stress exposure, the expression of GPx (Figure 1(a)) was markedly upregulated, and the highest RNA levels were observed in fish fed 400 mg/kg quercetin (*P* < 0.05). No marked effects were observed for GR (Figure 1(b)) and IL8 (Figure 1(c)), but they were higher in fish fed with quercetin-supplemented feeds (*P* > 0.05). The lysozyme gene expression was markedly higher in common carp fed with quercetin-supplemented feeds than in the control group (Figure 1(d)).

## 4. Discussion

In this study, the dietary incorporation of quercetin significantly improved growth and feed efficiency in common carp. Accordingly, the best WG, SGR, FI, and FCR were obtained in the fish fed with 400 and 600 mg/kg quercetin-supplemented diets. Previous findings indicated that dietary quercetin had a positive effect on the WG, SGR, and FCR in Nile tilapia (*Oreochromis niloticus*) [34], grass carp (*Ctenopharyngodon idella*) (), and common carp [13]. The promoting effect of quercetin on fish growth performance may be related to their better efficacy in stimulating the secretion of digestive enzymes like protease, amylase, and lipase [13, 34], decreasing the abundance of harmful bacteria of the intestine and promoting the populations of beneficial bacteria [35], ameliorating the intestinal barrier function, and improving the surface area of intestinal villi and goblet cells [36].

ACH50 activity is a significant parameter that benefitted in the evaluation of humoral nonspecific immune response in fish. A decrease in serum ACH50 levels is reported in different stress situations that fish are exposed to. In the present study, serum ACH50 activity significantly increased in the group supplemented with the doses of 400 and 600 mg/kg quercetin before the stress and after the stress compared to the control group. In conclusion, as the liver is the main source of complement proteins, the increase in ACH50 levels can be explained by improving the health and function of the liver. Our results in terms of increasing ACH50 activity are in full agreement with findings of earlier studies in common carp fed diets incorporated with 200 or 800 mg/kg quercetin [16]. Moreover, the results are in line with previous studies showing exogenous feed additives successful to prevent poststress complement activity reduction [37, 38].

Lysozyme is an important antimicrobial peptide of the innate immune system invading pathogens. A significant decrease in serum lysozyme activity was found in the control group of fish exposed at 32°C for 48 hours. A similar decrease in serum lysozyme activity at higher temperatures was observed in Nile tilapia, *Oreochromis niloticus* [39]. In the present study, serum lysozyme activity was significantly increased in all quercetin-supplemented groups before the stress and after the stress compared to the control group. This suggests that quercetin supplementation inhibits post-high-temperature stress immunosuppression.

Parallel with our study, lysozyme levels have also been increased in some fish species fed with quercetin-containing feeds. For instance, *C. carpio* fed with a diet containing 200 or 800 mg kg^−1^ quercetin showed an increase in lysozyme levels in serum, intestine, and mucus [16]. Wang et al. [18] added 0.01, 0.1, 1, 10, 100, and 1000 *μ*g/l quercetin to the rearing water of zebrafish (*Danio rerio*) and reported that serum lysozyme activity was significantly higher in the 1 *μ*g/l quercetin group than in the control group.

It is well known that serum glucose is used as a nonspecific stress index in fish studies [40, 41]. In this study, quercetin supplementations decreased serum glucose content significantly in the prestress group. Some reports are available on the hypoglycaemic effects of quercetin [42, 43]. This was supported in a recent study, where quercetin decreases serum glucose levels in common carp [16]. However, unlike our study, serum glucose levels in silver catfish, *Rhamdia quelen* [12], and blunt snout bream, *Megalobrama amblycephala* [44], fed with feed containing quercetin remained unchanged.

Superoxide dismutase (SOD), catalase (CAT), and glutathione peroxidase (GPx) are the main enzymes that scavenge reactive oxygen species (ROS) and detoxification of H_2_O_2_ and hydroperoxides, respectively. In addition, malondialdehyde (MDA) is frequently used as a biomarker of lipid peroxidation. Our results in terms of increasing SOD and decreasing MDA activity are in full agreement with findings of earlier studies in common carp, *C. carpio*, fed diets incorporated with 800 mg/kg quercetin. Similarly, Ibrahim et al. [45] reported the increased SOD and GPx and decreased MDA effect of quercetin in Nile tilapia, *Oreochromis niloticus*. Moreover, SOD mRNA expression [46] and brain, gill, liver, and/or muscle SOD and CAT activity levels [12] were higher in fish fed with quercetin diets. SOD, CAT, MDA, and GPx parameters play a significant role in antioxidant response in different stress conditions like pesticides [47], metals [48], high temperature [49, 50], hypoxia [50], and cold temperature [51]. However, no study is available on the effects of quercetin on antioxidant capacity in fish under high-temperature stress conditions. In the present study, decreased CAT, MDA, and GPx levels and increased SOD levels observed in post-high-temperature stress conditions indicate a significant antioxidant effect of quercetin. Similarly, the dietary anthraquinone extract especially at 0.1-0.2% levels significantly increasing SOD and decreasing MDA levels in the liver was reported in *M. rosenbergii* under high-temperature stress [25].

Serum enzyme activities are accepted as important indicators of tissue damage; for example, AST and ALT are indicators of liver damage and lactate dehydrogenase (LDH) for liver or muscle damage in fish [52]. Therefore, increases in these enzymes in blood could be attributed to liver and muscle tissue damage. Previous studies showed that the serum ALT and AST activities provided decreased when fish were fed with quercetin [13, 16]. No study on the effects of quercetin under high-temperature stress on fish serum enzyme levels has been found in the literature so far. However, [25] reported that AST and ALT activities in freshwater prawn (*M. rosenbergii*) fed a diet containing anthraquinone extract from *R. officinale* Bail under high-temperature stress were significantly lower than those of the control fish. In this study, serum AST and ALT (except for 200 mg/kg) decreased in fish fed with quercetin-supplemented feeds before high-temperature stress. This trend was sustained after high-temperature stress as well.

The low serum glucose levels in the high-temperature stressed fish may be the result of undernourished conditions, liver disorders, and inflammation [25, 50, 53]. This can be related to the increases in glucose use in peripheral tissues, probably related to the enhancement of energy demand generated by the stress response [54]. A similar reduction in the serum glucose level at a higher temperature (21°C) was observed in rainbow trout, *Oncorhynchus mykiss* [50]. In contrast to our findings, however, increased levels of glucose in blood were reported in Wuchang bream, *M. amblycephala*, under high-temperature stress [26]. These different results might be associated with differences in rearing conditions, exposure time, fish species, fish size, and/or age.

Blood cortisol is one of the most important indexes of the response to heat stress in fish [55]. In this study, cortisol levels were significantly lower in fish fed different quercetin levels before heat stress. Also, fish fed with diets supplemented with quercetin displayed significantly lower levels of cortisol after heat stress, highlighting the protective potential of quercetin supplementation on the metabolism of common carp. Similarly, Nile tilapia fed with dietary sodium butyrate showed a decrease in cortisol levels when exposed to heat stress [30]. In this study, cortisol was significantly lower during heat stress in fish fed quercetin as compared to the control group. These results may be related to antistress effects of quercetin that inhibited the domain of raised cortisol.

In line with these findings, it can be suggested that quercetin (400-600 mg/kg) may be a useful substance in the treatment and protection of cells against possible oxidative stress.

## Figures and Tables

**Table 1:** Experimental diets and proximate chemical composition.

Feedstuffs (g/kg)	Control	200	400	600
Fishmeal^1^	150	150	150	150
Meat meal^2^	200	200	200	200
Soybean meal	230	230	230	230
Wheat meal	339	338.8	338.6	338.4
Fish oil	7	7	7	7
Soybean oil	7	7	7	7
Corn flour	50	50	50	50
L-Lysine^3^	7	7	7	7
L-Methionine 100^3^	5	5	5	5
Vitamin premix^4^	2.5	2.5	2.5	2.5
Mineral premix^5^	2.5	2.5	2.5	2.5
Quercetin (mg/kg)	0	0.2	0.4	0.6

Chemical composition				
Gross energy (kcal kg^−1^)	4097.04	4096.25	4095.47	4094.68
Dry matter (%)	86.22	86.20	86.18	86.16
Crude protein (%)	38.21	38.21	38.21	38.20
Crude fat (%)	6.23	6.23	6.23	6.23
Crude ash (%)	6.37	6.37	6.36	6.36

^1^Pars Kilka Co., Mazandaran, Iran (kilka powder analysis; protein: 70-72%, fat: 8-11%, ash: 11.6%, and moisture: 7-9%). ^2^Makianmehr Co., Golestan, Iran. ^3^Morghenojan.Co, Tehran, Iran. ^4^Vitamin premix (per kg of diet): vitamin A: 2000 IU; vitamin B_1_ (thiamin): 5 mg; vitamin B_2_ (riboflavin): 5 mg; vitamin B_6_: 5 mg; vitamin B_12_: 0.025 mg; vitamin D_3_: 1200 IU; vitamin E: 63 mg; vitamin K_3_: 2.5 mg; folic acid: 1.3 mg; biotin: 0.05 mg; pantothenic acid calcium: 20 mg; inositol: 60 mg; ascorbic acid (35%): 110 mg; niacinamide: 25 mg. ^5^Mineral premix (per kg of diet): MnSO_4_: 10 mg; MgSO_4_: 10 mg; KCl: 95 mg; NaCl: 165 mg; ZnSO_4_: 20 mg; KI: 1 mg; CuSO_4_: 12.5 mg; FeSO_4_: 105 mg; Co: 1.5 mg.

**Table 2:** Primers selected for the expression of the selected genes.

Gene	Primer	Fragment length (bp)	Accession no.
IL8	F: 5′ CTTCACTGGTGTTGCTCTGC 3′	20	XM_019080796.1
	R: 5′ TGTTGGCTTTTAGGTCTACCC 3′	21	
Lysozyme	F: 5′ CCACTCTCCATCTGAACTCC 3′	20	XM_019104788.1
	R: 5′ ACATCAGACACAAGACAGCAA 3′	21	
GPx	F: 5′ CAACAGGAGAATGCCAAGA 3′	19	XM_019093635.1
	R: 5′ AGGAACACGAACAGAGGGT 3′	19	
GR	R: 5′ CGCCAATCACCAGAAAATCA 3′	20	XM_042770144.1
	F: 5′ CACAAACGAGGACAAGACCA 3′	20	
GAPDH	F: 5′ CACAAACGAGGACAAGACCA 3′	20	XM_019119762.1
	R: 5′ CCTCACCCTTGTACTTTCCA 3′	20	

**Table 3:** Growth performance and feed efficiency of common carp fed with experimental diets after 60 days.

Growth performance	Control	200	400	600
IW (g)	27.36 ± 1.22	26.90 ± 1.26	27.53 ± 1.43	27.08 ± 1.18
FW (g)	57.25 ± 3.35^b^	59.11 ± 3.99^b^	65.17 ± 3.73^a^	66.94 ± 5.95^a^
WG (g)^1^	29.88 ± 3.79^b^	32.35 ± 4.33^b^	37.64 ± 3.87^a^	39.86 ± 5.99^a^
WG (%)^2^	109.68 ± 16.56^b^	120.81 ± 19.76^b^	137.24 ± 17.42^a^	147.53 ± 23.36^a^
SGR (%/d)^3^	1.22 ± 0.13^b^	1.31 ± 0.14^b^	1.43 ± 0.12^a^	1.50 ± 0.16^a^
FI (g)	54.41 ± 1.47^b^	52.56 ± 1.77^c^	56.02 ± 1.49^a^	57.266 ± 2.06^a^
FCR^4^	1.85 ± 0.28^a^	1.65 ± 0.21^b^	1.50 ± 0.16^b^	1.46 ± 0.22^b^
SR^5^	100	100	100	100

^1^Weight gain (WG, g) = final weight − initial weight. ^2^Weight gain (WG, %) = 100 × ((final weight − initial weight)/initial weight). ^3^Specific growth rate (SGR) = ((Ln W2 − Ln W1)/During the total experimental period (56 days)) × 100. ^4^Feed Conversion Ratio (FCR) = Dry feed consumed (g)/WG (g). ^5^Survival (SR, %) = (Number of fish in each group remaining in end of feeding trial/initial number of fish) × 100. Different letters designate significant differences as determined by Duncan's multiple range test (*P* < 0.05).

**Table 4:** Immune response of common carp fed different levels of quercetin for 60 days before and after heat stress.

Experiment groups	Parameters	
	ACH50 (U %)	Lysozyme (U/ml/min)
Before stress		
0	133.76 ± 0.44^cC^	30.62 ± 0.45^bB^
200	134.09 ± 0.95^cC^	33.92 ± 1.46^aA^
400	140.82 ± 1.69^aA^	35.89 ± 1.23^aA^
600	138.10 ± 1.04^bAB^	35.01 ± 2.47^aA^

After stress		
0	128.30 ± 3.04^cD^	22.94 ± 1.33^cD^
200	130.78 ± 0.78^bcD^	26.39 ± 0.98^bC^
400	136.58 ± 2.48^aBC^	27.76 ± 0.32^abC^
600	134.19 ± 1.02^abC^	28.31 ± 0.72^aC^

Two-way ANOVA		
Stress	*P* < 0.001	*P* < 0.001
Diet	*P* < 0.001	*P* < 0.001
Interaction	NS	NS

Complement pathway activity (ACH50). Different lowercase superscript letters within the same column mean significantly different in each group separately before the heat challenge and after the heat challenge. Different uppercase superscript letters within the same column indicated a significant difference in all means with each other before and after the heat challenge.

**Table 5:** Antioxidant enzymes of common carp fed different levels of quercetin for 60 days before and after heat stress.

Experiment groups	Parameters			
	CAT (U/ml)	SOD (U/ml)	MDA (*μ*mol/l)	GPx (U/ml)
Before stress				
0	88.12 ± 0.97^aC^	52.12 ± 1.25^cD^	95.57 ± 1.70^aD^	166.04 ± 2.92^aD^
200	82.71 ± 1.54^bD^	62.19 ± 0.95^bB^	89.14 ± 1.99^bE^	155.66 ± 0.31^bE^
400	66.39 ± 1.49^dF^	67.48 ± 1.52^aA^	81.36 ± 2.43^cF^	164.10 ± 3.99^aD^
600	71.63 ± 1.71^cE^	67.14 ± 1.86^aA^	90.58 ± 1.37^bE^	149.60 ± 1.46^cF^

After stress				
0	96.14 ± 0.77^aA^	43.08 ± 1.94^dF^	109.33 ± 2.94^aA^	186.48 ± 3.60^bB^
200	95.72 ± 2.79^abA^	47.16 ± 2.17^cE^	103.77 ± 0.47^bB^	211.78 ± 0.80^aA^
400	91.50 ± 5.35^bB^	57.64 ± 1.27^aC^	98.99 ± 0.19^cC^	174.66 ± 1.58^cC^
600	93.02 ± 2.86^abAB^	53.59 ± 1.77^bD^	105.72 ± 2.71^abB^	167.15 ± 2.34^dD^

Two-way ANOVA				
Stress	*P* < 0.001	*P* < 0.001	*P* < 0.001	*P* < 0.001
Diet	*P* < 0.001	*P* < 0.001	*P* < 0.001	*P* < 0.001
Interaction	*P* < 0.001	*P* = 0.016	NS	*P* < 0.001

CAT: catalase; SOD: superoxide dismutase; MDA: malondialdehyde; GPx: glutathione peroxidase. Different lowercase superscript letters within the same column mean a significant difference in each group separately before the heat challenge and after the heat challenge. Different uppercase superscript letters within the same column indicated a significant difference in all means with each other before and after the heat challenge.

**Table 6:** Stress factors of common carp fed different levels of quercetin for 60 days before and after heat stress.

Experiment groups	Glucose (mg/dl)	Cortisol (ng/ml)	AST (U/l)	ALT (U/l)
Before stress				
0	104.16 ± 1.14^aA^	99.87 ± 0.36^aA^	191.91 ± 6.08^aCD^	13.40 ± 0.75^aBC^
200	64.81 ± 1.20^bE^	89.45 ± 4.47^bBC^	169.32 ± 3.85^bF^	12.07 ± 1.79^abCD^
400	55.55 ± 3.90^cF^	75.20 ± 1.88^cD^	179.37 ± 6.85^bE^	11.20 ± 0.85^bD^
600	59.15 ± 1.93^cF^	85.62 ± 1.55^bC^	145.33 ± 6.29^cG^	10.69 ± 0.31^bD^

After stress				
0	98.86 ± 0.53^aB^	101.29 ± 2.93^aA^	246.94 ± 5.98^aA^	16.02 ± 0.98^aA^
200	94.92 ± 0.44^aB^	90.39 ± 0.89^bcBC^	204.41 ± 4.57^bB^	14.35 ± 0.56^bB^
400	71.64 ± 4.55^cD^	86.27 ± 4.83^cC^	200.03 ± 1.73^bBC^	14.54 ± 0.42^bAB^
600	81.23 ± 1.86^bC^	92.80 ± 0.78^bB^	190.27 ± 1.36^cD^	13.64 ± 0.32^bBC^

Two-way ANOVA				
Stress	*P* < 0.001	*P* < 0.001	*P* < 0.001	*P* < 0.001
Diet	*P* < 0.001	*P* < 0.001	*P* < 0.001	*P* = 0.001
Interaction	*P* < 0.001	*P* = 0.015	*P* < 0.001	NS

AST: aspartate aminotransferase; ALT: alanine aminotransferase. Different lowercase superscript letters within the same column mean a significant difference in each group separately before the heat challenge and after the heat challenge. Different uppercase superscript letters within the same column indicated a significant difference in all means with each other before and after the heat challenge.

## Data Availability

The datasets generated during and/or analyzed during the current study are available from the corresponding authors on reasonable request.
